# Successful management of left-sided thoracic impalement in a resource-limited setting: A case report

**DOI:** 10.1016/j.ijscr.2025.111310

**Published:** 2025-04-17

**Authors:** Suleiman Ayalew Belay, Michael A. Negussie, Yirgedu Getu Tadele, Ashenafi Berhanu Adale, Samrawit Andarge Kassa, Muluken Assefa Zemariam

**Affiliations:** a School of Medicine, College of Medicine and Health Sciences, University of Gondar, Gondar, Ethiopia; b School of Medicine, College of Health Sciences, Addis Ababa University, Addis Ababa, Ethiopia; c Department of Surgery, School of Medicine, College of Medicine and Health Sciences, University of Gondar, Gondar, Ethiopia

**Keywords:** Thoracic impalement, Penetrating chest trauma, Foreign body removal, Resource-limited setting, Case report

## Abstract

**Introduction:**

Thoracic impalement injuries are rare and life-threatening. Managing them in resource-limited settings poses significant challenges.

**Case presentation:**

A 63-year-old male fell 3 m from a tree, sustaining a left-sided chest impalement at the 7th intercostal space along the mid-axillary line. On arrival, his vitals were BP 100/70 mmHg, pulse 104 bpm, RR 32/min, and SpO₂ 78 % (improving to 90 % with oxygen). Examination revealed absent air entry on the left, a sucking chest wound, and an impaled wooden fragment. Initial management included resuscitation, antibiotics, and tetanus prophylaxis. A left anterolateral thoracotomy revealed 7th and 8th rib fractures, lung damage, and an embedded wooden object, which was removed with wedge resection and chest tube placement. The patient had an uneventful recovery and was discharged in stable condition.

**Discussion:**

Thoracic impalement injuries pose significant challenges due to the risk of severe hemorrhage, infection, and organ damage. Left-sided injuries are particularly critical due to proximity to the heart and great vessels. Avoiding premature removal of the foreign object, prioritizing hemodynamic stabilization, and expediting surgical intervention are key principles in management.

**Conclusion:**

Timely intervention, controlled foreign body removal, and adherence to trauma protocols are essential for successful management of thoracic impalement injuries, even in resource-limited settings.

## Highlights

• Thoracic impalement injuries are rare and life-threatening.
• A 63-year-old male fell onto a wooden object, causing a left-sided thoracic impalement with lung and rib injuries.
• The wooden object was surgically removed via thoracotomy, avoiding major vascular or airway injury.
• Postoperative infection and effusion were managed with antibiotics, drainage, and ultrasound-guided aspiration.
• Trauma protocols, controlled foreign body removal, and multidisciplinary care ensured a successful outcome.

## 1 Introduction

Impalement injuries involve the penetration of a body cavity or extremity by a large foreign object, such as a steel bar or wooden item, which remains embedded in the body [1]. Thoracic impalement injuries are rare in civilian populations and often present dramatically [2]. This report details the case of a 63-year-old male who survived a left chest impalement by a large wooden object following a fall.

This case has been reported in accordance with the SCARE criteria [3].

## 2 Case presentation

**Fig. 1:**
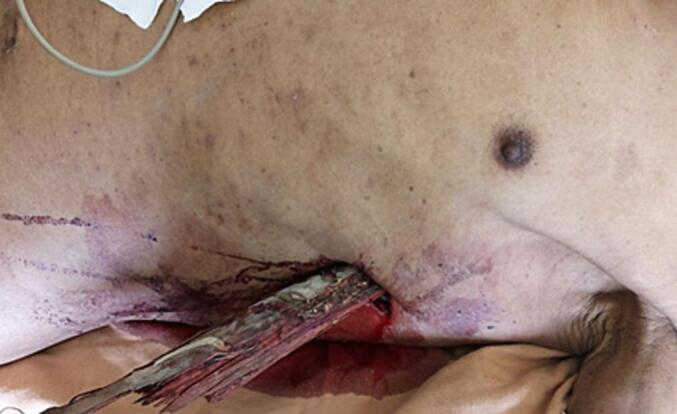
Preoperative image of the impaled wooden object.

A 63-year-old male farmer with no history of smoking presented to Gondar Specialized Hospital (a tertiary care center in Ethiopia) with a chest impalement injury caused by a wooden object. The incident occurred when he fell approximately 3 m from a tree onto a wooded surface. The wood penetrated his left chest at the 7th intercostal space along the mid-axillary line, with a medial and upward trajectory (Fig. 1). The patient reported chest pain, shortness of breath, and bleeding from the wound.

He arrived at the hospital 4 h post-injury. On admission, his blood pressure was 100/70 mmHg, pulse rate was 104 beats per minute, respiratory rate was 32 breaths per minute, and oxygen saturation was 78 % on room air, improving to 90 % with a face mask. Examination revealed absent air entry in the left two-thirds of the lung field, a sucking chest wound, and the impaled wooden object. The patient remained conscious with a Glasgow coma scale score of 15. No other traumatic injuries were identified, and his past medical history was unremarkable.

Broad-spectrum antibiotics, including ceftriaxone and metronidazole, along with tetanus antitoxin, were administered, and an emergency thoracotomy was scheduled. Under general anesthesia with endotracheal intubation, the patient was positioned supine with a slight right tilt. A left anterolateral thoracotomy was performed using aseptic techniques.

**Fig. 2:**
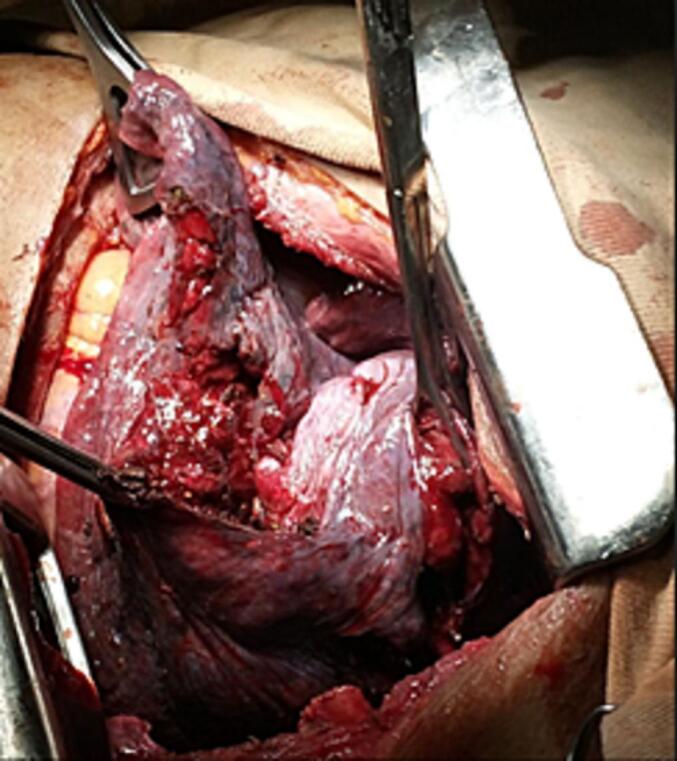
Intraoperative image showing left lung parenchymal injury.

Intraoperatively, a 6 cm round wooden object was found penetrating the chest, fracturing the 7th and 8th ribs laterally. The object had pierced through the lower lobe of the left lung, exiting near the apex close to the left subclavian vessels without injuring major vessels or airways. Extensive lung parenchymal damage was observed (Fig. 2).

The wooden object was carefully removed under direct vision. A wedge resection of the damaged lung tissue was performed, hemostasis was achieved, and the thoracic cavity was lavaged with saline. A chest tube was placed, and the incision was closed in layers. The patient was transferred to the intensive care unit (ICU) for postoperative monitoring.

**Fig. 3:**
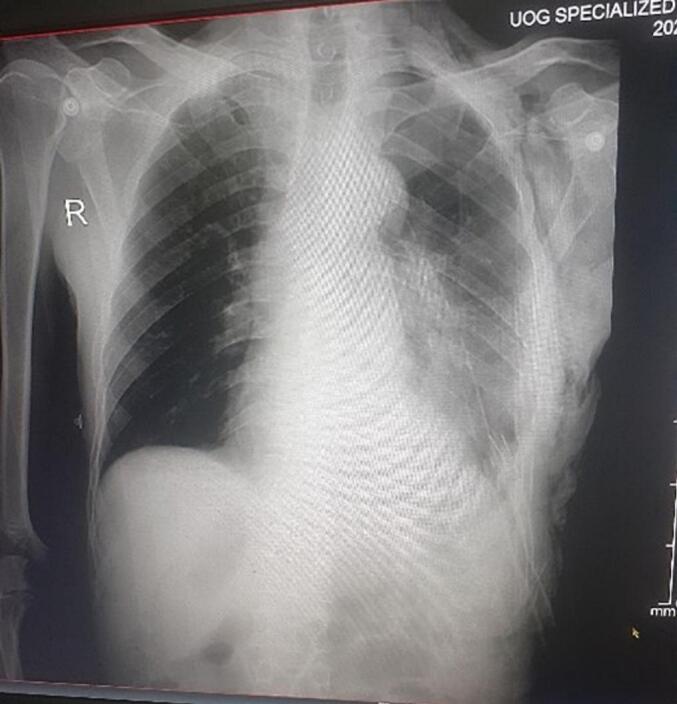
Postoperative chest X-ray on day 2 showing lung expansion.

The patient remained in the ICU for three days before being transferred to the surgical ward. A chest X-ray performed on postoperative day 2 (Fig. 3) showed a well-expanded left lung. On the fifth day of admission, the patient developed left-sided chest pain, low-grade fever, and purulent drainage from the chest tube. A complete blood count revealed leukocytosis**.** A follow-up chest X-ray subsequently demonstrated a significant left-sided fluid collection.

The chest tube was manipulated, the antibiotic regimen was revised to include vancomycin and cefepime, which were administered for 15 days, and the tube was irrigated with ciprofloxacin. By postoperative day 13, the chest tube was removed following the resolution of drainage (no output for 72 h) and ultrasound findings demonstrating only parenchymal consolidation with minimal pleural fluid. A chest X-ray on the 13th day and an ultrasound on the 14th day showed a 1.4 cm anechoic collection with consolidation, which was tapped under ultrasound guidance, yielding clear serous fluid. The patient was discharged after a 20-day hospital stay and was in good condition at his two-week follow-up.

Long-term follow-up at 1, 3, and 6 months confirmed the durability of the surgical outcome, with no late sequelae observed.

## 3 Discussion

Civilian thoracic impalement injuries typically result from falls onto sharp objects, motor vehicle accidents, or assaults involving projectiles [4]. These injuries are complex, often involving crushing, penetration, tissue loss, contamination, fractures, and significant hemorrhage, posing substantial challenges to surgical teams [5].

Impalement injuries are broadly categorized into two types: Type I injuries occur when a moving body strikes a stationary object, such as in falls or motor vehicle collisions, while Type II injuries result from a moving object piercing a stationary body, as seen in assaults or projectile injuries [6]. Our case represents a Type I injury caused by a fall onto a wooden object. Cardiac injury was excluded by stable hemodynamics and absence of clinical signs of tamponade. Tension pneumothorax was ruled out by absent tracheal deviation and maintained oxygenation.

Survival rates are higher for right-sided thoracic injuries due to the lower risk of cardiac and great vessel involvement [7]. Left-sided impalements, like in this case, are rarer and often more severe. Despite the wooden object penetrating the lung, our patient had no major vessel or airway injury, contributing to his favorable outcome [8].

Management of impalement injuries requires transporting the patient with the foreign body in situ and avoiding removal outside a controlled surgical setting [1,11]. Initial evaluation should follow the Advanced Trauma Life Support (ATLS) protocol [2,11]. Surgical exploration by a multidisciplinary team, including cardiothoracic, gastrointestinal, orthopedic, and plastic surgeons, is often necessary [9]. In cases of major vascular injury, cardiopulmonary bypass may be required [12,13].

Radiological investigations should be minimized in hemodynamically unstable patients to avoid delays and potential further injury from object movement [10]. In this case, no preoperative imaging was performed to expedite surgical intervention.

In well-resourced settings, stable patients with thoracic impalement injuries often undergo preoperative contrast-enhanced CT scans to map the path of the object, identify injuries to blood vessels or organs, and guide surgical planning. In our case, no imaging was performed before surgery. This was partly due to limited access to advanced imaging and partly because of the urgency of the situation. Delaying surgery for imaging could have increased the risk of complications. The decision to proceed directly to thoracotomy was based on clinical assessment, stable vital signs, and the visible presence of a large foreign object that could have caused further harm if disturbed. After surgery, chest X-rays and bedside ultrasound were used to monitor recovery and guide further management. This case illustrates that even without advanced imaging, timely decision-making based on sound clinical judgment can lead to successful outcomes in resource-limited settings.

## 4 Conclusion

Left-sided thoracic impalement injuries are rare and often fatal if not managed promptly. Successful outcomes depend on swift and coordinated multidisciplinary care, adherence to trauma management protocols, and careful intraoperative technique.

## Consent for publication

Written informed consent was obtained from the patient for publication of this case report and accompanying images. A copy of the consent is available upon request.

## Ethical approval

Ethical approval was obtained for the publication of this case report.

## Funding

No funding was received for this case report.

## Author contribution

**Suleiman Ayalew Belay**: Writing - original draft, Data curation, Conceptualization, Resources.

**Michael A. Negussie**: Writing - review and editing, Conceptualization.

**Yirgedu Getu Tadele**: Writing - original draft, Resources.

**ASHENAFI BERHANU ADALE**: Data curation, Conceptualization.

**Samrawit Andarge Kassa:** Writing - review and editing, Conceptualization.

**Muluken Assefa Zemariam:** Data curation, Conceptualization.

## Guarantor

Dr. Suleiman Ayalew Belay.

## Declaration of competing interest

The authors declare that they have no known competing financial interests or personal relationships that could have appeared to influence the work reported in this paper.
